# Bereavement care in UK ICUs: a national survey

**DOI:** 10.1186/cc14658

**Published:** 2015-03-16

**Authors:** M Berry, E Brink, V Metaxa

**Affiliations:** 1 Imperial Healthcare Trust, London, UK; 2 King's College Hospital, London, UK

## Introduction

For the families of critically ill patients, the death of a loved one in the ICU is often an unexpected and traumatic event, characterised by difficult decisions regarding withholding or withdrawing life-sustaining therapy. Increasingly the importance of bereavement care (BC) in the ICU is being acknowledged, although reports continue to highlight the inadequacies around end-of life care in the critical care environment. In 1998, the Intensive Care Society (ICS) published guidelines mapping out BC in the ICU [1]. We aimed to compare BC in ICUs across England against the recommendations set out by the ICS.

## Methods

All adult ICUs in England were contacted over a 2-week period, using a standardised questionnaire based on the nine domains identified by the ICS. All answers were collected anonymously using SurveyMonkey®. An 80% compliance rate was deemed acceptable.

## Results

From the 148 ICUs identified, 113 answered the questionnaire (76%). Forty-three per cent of the responders had access to training in BC and in communication skills, and 54% had a named member of staff responsible for training, writing, auditing and developing the BC policy. When asked about the presence of a written BC policy only 45% responded positively, and even less (19%) had provisions for audit and development of the service. Information to staff about cultural and religious rites around the time of death, and to relatives on what to do after a death was available in 81% and 96% respectively. The general practitioner was informed of the deaths taking place in the ICU in 77% of the cases. In more than 70% of the participating ICUs, efforts were made to ensure privacy of the grieving relatives and to have dedicated follow-up facilities for the bereaved. Even though staff support programmes were recognised as paramount, only 54% of the ICUs had formal ones set up.

## Conclusion

This is the first national audit of BC in the ICU since the initial ICS guideline publication. Even though most ICUs provided relatives with information around the time of death, training, auditing and adequate facilities do not meet the recommended standards. The lack of adherence is definitely multifactorial and requires further research. In the meantime, vigorous implementation of these guidelines is warranted in order to ensure optimal care for the bereaved families.
